# miRNAs and exosomes in psoriasis: coordinating cytoskeleton dynamics and extracellular matrix remodeling

**DOI:** 10.3389/fcell.2025.1608902

**Published:** 2025-07-18

**Authors:** Sijing Li, Zamri Chik, Farid Nazer Faruqu, Najihah Mohd Hashim, Nor Saadah Mohd Yusof, Jennifer Fernandez Alarcon, Noraini Ahmad

**Affiliations:** ^1^ Department of Chemistry, Faculty of Science, Universiti Malaya, Kuala Lumpur, Malaysia; ^2^ Department of Biomedical Engineering, School of Engineering, Dali University, Dali, Yunnan, China; ^3^ Universiti Malaya Research Centre for Biopharmaceuticals and Advanced Therapeutics (UBAT), Department of Pharmacology, Faculty of Medicine, Universiti Malaya, Kuala Lumpur, Malaysia; ^4^ Department of Pharmaceutical Chemistry, Faculty of Pharmacy, Universiti Malaya, Kuala Lumpur, Malaysia; ^5^ Grup d'Enginyeria de Materials (Gemat), Institut Químic de Sarrià (IQS), Universitat Ramon Llull (URL), Barcelona, Spain

**Keywords:** psoriasis, miRNA, exosome, cytoskeleton, extracellular matrix

## Abstract

Psoriasis is a chronic inflammatory skin disorder characterized by hyperproliferation of keratinocytes, immune dysregulation, and abnormal epidermal differentiation. Its pathogenesis involves complex interactions among keratinocytes, fibroblasts, T cells, and myeloid cells, where dynamic cytoskeletal and extracellular matrix changes critically mediate intercellular communication. Emerging evidence highlights the pivotal roles of miRNAs and exosomes in coordinating these processes: miRNAs regulate cytoskeletal organization and extracellular matrix composition, while exosomes act as intercellular messengers that deliver miRNA-mediated signals, collectively shaping cell behavior and disease progression. This review synthesizes current knowledge on how miRNA-exosome networks drive cytoskeleton-extracellular matrix crosstalk in psoriasis, emphasizing their implications for cellular communication and tissue remodeling. By elucidating these mechanisms, we identify potential therapeutic opportunities to target pathogenic signaling pathways, offering new strategies for psoriasis management.

## 1 Introduction

Psoriasis is a chronic, immune-mediated inflammatory skin disease that is caused by the rapid proliferation and abnormal differentiation of keratinocytes, leading to the generation of thick, squamous plaques on the skin (Armstrong and Read, 2020). This disease affects approximately 2%–3% of the global population, making it a significant health concern because of its impact on patients’ quality of life and its association with diverse comorbidities, including cardiovascular disorders, metabolic diseases, and depression (Wolk and Sabat, 2016; Sugumaran et al., 2024).

The pathogenesis of psoriasis stems from genetic predisposition (Chandra et al., 2015), environmental triggers, and immune dysregulation, mediated through complex cellular interactions (Hussain et al., 2022). While the keratinocyte-immune cell axis remains central (Armstrong and Read, 2020; Zhou et al., 2022), emerging single-cell evidence reveals critical fibroblast involvement (Ma et al., 2023). These ECM-remodeling cells participate in miRNA-mediated regulation through distinct mechanisms. Both intracellular and exosomal miRNAs from these cell types contribute to ECM dysregulation and cytoskeletal dysfunction, creating a self-perpetuating cycle of inflammation. For example, intracellular miRNAs, such as miR-203 in keratinocytes, directly modulate cytoskeletal organization, whereas secreted miRNAs - including fibroblast-derived exosomal miR-21 - exert paracrine effects on neighboring keratinocyte differentiation (Jevtić et al., 2020; Ma et al., 2023). This establishes a pathological feedback loop wherein ECM alterations (Ghaffari et al., 2006; Philips et al., 2009) both originate from and reinforce dysregulated miRNA signaling across cell populations (Clément et al., 2022). Therapeutic interventions targeting psoriasis must consequently address both cell-autonomous miRNA functions and intercellular miRNA communication networks. Moreover, Understanding the interactions among fibroblasts, keratinocytes, and the ECM is crucial for developing targeted therapies that aim to normalize these interactions and restore a healthy skin architecture.

The cytoskeleton and ECM are fundamental to the structural and functional integrity of skin cells, particularly keratinocytes and fibroblasts, which play central roles in the pathological process of psoriasis (Ghaffari et al., 2006; Philips et al., 2009; Jalal et al., 2019). By studying the cytoskeleton and ECM in psoriasis, potential therapeutic targets can be identified. For example, modulating the activity of miRNAs, proteins and exosomes involved in cytoskeletal organization or ECM remodeling could help restore normal cell functions and reduce inflammation. Additionally, therapies aimed at normalizing the ECM composition or enhancing ECM repair could improve skin barrier function and reduce the severity of psoriatic lesions (Glinski et al., 1995; Holm Nielsen et al., 2023).

This comprehensive review elucidates the critical roles of miRNA and exosome-mediated communication in orchestrating cytoskeletal reorganization and ECM remodeling during psoriatic pathogenesis, highlighting how specific miRNA families differentially regulate both cytoskeletal dynamics in keratinocytes/immune cells and key ECM components of fibroblasts. The emerging paradigm reveals psoriatic exosomes as sophisticated carriers that transport regulatory miRNAs along with cytoskeletal proteins and ECM-modifying enzymes, exhibiting distinct biophysical properties that enhance pathological intercellular communication. These mechanistic insights provide a unified framework for understanding epithelial-immune dysregulation while offering novel diagnostic biomarkers and multiple therapeutic targets, ranging from miRNA-based therapies to engineered exosomes that normalize cellular mechanics and matrix homeostasis. By bridging current knowledge gaps and proposing a mechanobiology-oriented treatment approach, this review not only advances psoriasis research but also establishes conceptual frameworks applicable to other fibroproliferative and inflammatory disorders characterized by similar cytoskeletal-ECM dysregulation.

### 1.1 Summary of psoriasis’s pathogenesis

**FIGURE 1:**
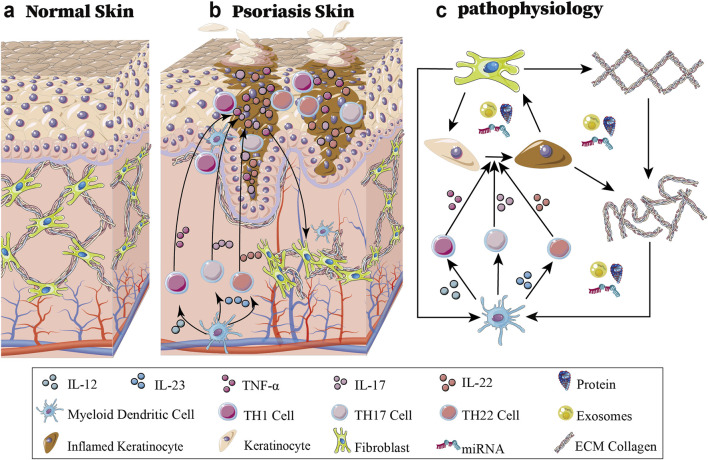
Schematic diagram of normal skin and psoriatic skin and the associated pathophysiology. **(a)** Shows normal skin architecture. **(b)** Shows psoriatic lesion demonstrating altered cellular distribution and interactions between immune cells (dendritic cells, T helper subsets), hyperproliferative keratinocytes, and activated fibroblasts. **(c)** Demonstrates the immunopathogenic cascade: myeloid dendritic cell-derived IL-12/IL-23 initiates T cell differentiation into TH1 (TNF-α), TH17 (IL-17), and TH22 (IL-22) subsets, driving keratinocyte hyperproliferation. This inflammatory milieu promotes pathogenic exosome release containing regulatory miRNAs/proteins that remodel the extracellular matrix and reprogram fibroblasts, which in turn further amplify immune activation, establishing a self-perpetuating inflammatory cycle central to psoriatic plaque formation and maintenance.

Psoriasis is a complex disease involving diverse interactions among genetic, environmental, and immunological factors. Aberrant activation of signaling pathways and dysregulated cellular processes contribute to the pathogenesis of psoriasis. The adaptive immune system plays important roles in pathophysiologies that involve immoderate feed-forwards immune activation (Armstrong and Read, 2020; Owczarczyk-Saczonek et al., 2020). IL12 and IL23 are produced in large amounts by activated myeloid dendritic cells. Naive T cells differentiate into T helper type 1 (TH1) cells due to the influence of IL12 overexpression. TH1 cells secrete tumour necrosis factor α (TNF-α). TH17 and TH22 cells can survive and proliferate in response to the growth factor IL23. IL17 is secreted by a variety of inflammatory cells, such as TH17 cells, and IL22 can also be produced by TH22 cells (Chen et al., 2021). In fact, various cell types, not only immune cells but also structural cells, such as keratinocytes and fibroblasts, are associated with psoriasis development and play vital roles in this process (Gliński et al., 1993; Conrad et al., 2007; Tervaniemi et al., 2018; Castro-Manrreza et al., 2019; Grivas et al., 2022). Signal transduction inside keratinocytes is activated by these secreted cytokines, resulting in the transcription of cytokines and chemokines, especially the IL-23/IL-17 axis via STAT3, TNF-α/NF-κB, EGFR-MAPK cascade, and Notch/Wnt-β-catenin signaling, resulting in the transcription of cytokines (IL-1β, IL-6, IL-8, IL-36γ, TNF-α), chemokines (CXCL1/2/8/9/10, CCL20) and growth factors (VEGF, FGF7, TGF-α). The resulting inflammatory cascade stimulates fibroblast recruitment through IL-1β/TNF-α-mediated chemotaxis, while keratinocyte-derived TGF-β promotes excessive collagen I/III production. Concurrent MMP-9 overexpression degrades normal ECM components, establishing a fibronectin-rich pathological matrix that perpetuates inflammation and facilitates abnormal keratinocyte proliferation, thereby creating a self-sustaining inflammatory-proliferative loop characteristic of psoriatic lesions (Chandra et al., 2015; Pfisterer et al., 2021; Wagner et al., 2021). Additionally, type 1 Interferon (IFN), IFNG and TNF promote the transformation of fibroblasts from a profibrotic state to a primary inflammatory state (Ma et al., 2023), and the ECM becomes more irregular (see Figure 1).

An altered spatial distribution of keratinocytes and fibroblasts can lead to psoriatic disease manifestations. During these processes, the cytoskeleton and ECM participate in maintaining tissue integrity and regulating cell behavior. Emerging evidence suggests that miRNAs and exosomes are essential participants in controlling the dynamic interplay between these cellular components (Ge et al., 2014; Hawkes et al., 2016; Jiang et al., 2019; 2023; Solvin et al., 2022; Iuliano et al., 2024).

Abnormal production of miRNAs or exosomes in keratinocytes, fibroblasts, T cells and myeloid cells contributes to the onset of early psoriasis symptoms, and increased accumulation of these factors results in qualitative changes (Ghaffari et al., 2006; Conrad et al., 2007; Wcisło-Dziadecka et al., 2018; Jalal et al., 2019; Jevtić et al., 2020; Nasiri et al., 2020; Clément et al., 2022; Ma et al., 2023; Chakraborty et al., 2016). In his process, the posttranscriptional regulation of miRNAs, proteins and miRNAs in exosomes, which mediate remote signal transmission, results in different levels of competition and cooperation at the molecular, cell, and tissue levels. Dynamic changes to maintain homeostasis can keep the skin healthy, but when homeostasis is disrupted, diseases can occur (Haensel et al., 2020).

### 1.2 Role of cytoskeleton remodeling in the pathogenies of psoriasis

The cytoskeleton, which consists of actin filaments, microtubules, and intermediate filaments, is essential for maintaining the cell shape, allowing cellular movement, and promoting intracellular transport (Sotodosos-Alonso et al., 2023). In psoriasis, abnormalities in the keratinocyte cytoskeleton have been observed, and these changes can lead to keratinocyte hyperproliferation and impaired differentiation. This results in the characteristic thick, scaly plaques observed in psoriatic lesions (Tervaniemi et al., 2012; Tervaniemi et al., 2018; Zhang et al., 2023). Disrupted cytoskeletal organization can also affect cell signaling pathways that regulate inflammation and immune responses, exacerbating the disease state (Gliński et al., 1993; Tervaniemi et al., 2018).

The cytoskeleton plays a pivotal role in intracellular transport and cellular organization, processes that are fundamentally disrupted in psoriasis pathogenesis (Vale, 2003; Lin et al., 2022; Zhou et al., 2022). Microtubules, the largest filaments, not only mediate organelle and vesicle trafficking through kinesin and dynein motor proteins, but their dysregulated dynamics of protein light chain three due to higher expression of CYP1A1 and AHR in psoriatic keratinocytes contribute to abnormal cell polarization and disrupted cytokine secretion (Vale, 2003; Kim et al., 2021; Lo et al., 2021). Notably, in psoriasis lesions, altered microtubule networks impair the proper trafficking of key inflammatory mediators and contribute to the hyperproliferative phenotype characteristic of this disease (Chen et al., 2021; Lo et al., 2021). Actin filaments, through their interaction with myosin motors, facilitate vesicular transport and are critically involved in the pathological cell migration observed in psoriatic epidermis with high concentration of IL-9 and IL-17. Their reorganization drives both keratinocyte hyperproliferation and the aberrant immune cell infiltration that sustains chronic inflammation (Vale, 2003; Das et al., 2019). Intermediate filaments primarily provide structural support, their characteristic dysregulation in psoriatic skin, particularly keratin mutations (Keratin 6, 16 and 17), directly compromises cell integrity and indirectly exacerbates transport defects by destabilizing the overall cytoskeletal architecture (McKay and Leigh, 1995; Vale, 2003; Zhang et al., 2019). Based on the knowledge of synergy between cytoskeleton (Vale, 2003), the coordinated remodeling of all three filament systems in psoriasis may create a self-amplifying cycle: cytoskeletal alterations promote inflammatory signaling, which in turn induces further cytoskeletal reorganization, perpetuating disease progression. This cytoskeletal dysregulation extends beyond intracellular effects, as disrupted filament networks in psoriatic cells markedly influence extracellular matrix deposition and remodeling through aberrant exosome secretion and growth factor trafficking, thereby contributing to the characteristic dermal-epidermal alterations in psoriasis (Bhattacharjee et al., 2019).

### 1.3 Role of extracellular matrix remodeling in the pathogenesis of psoriasis

The ECM is a network of multiple proteins and polysaccharides that provides structural assistance to tissues and regulates various cellular functions, including adhesion, migration, and proliferation (Karamanos et al., 2021; Pfisterer et al., 2021; Wagner et al., 2021; Doyle et al., 2022). In psoriatic skin, there are significant alterations in ECM components, including collagen, fibronectin, and laminin (Pfisterer et al., 2021; Wagner et al., 2021). These changes can disrupt the normal structure of the skin and impair its barrier function. Moreover, the ECM interacts with receptors on the cell surface to stimulate cell behavior and tissue remodeling (Pfisterer et al., 2021; Wagner et al., 2021). In psoriasis, dysregulation of ECM components like α1β1 integrin, dystroglycans, and toll-like receptors and their interactions with cells can perpetuate inflammation and abnormal tissue growth (Conrad et al., 2007; Mezentsev et al., 2014; Pfisterer et al., 2021; Wagner et al., 2021).

Different types of cells within a tissue have unique spatial distributions, which support their specific functions. There are spatial boundaries and fixed topological structures associated with different cells (Karamanos et al., 2021; Doyle et al., 2022). When the structure of a material changes, its macroscopic shape and biological function also change. Different cells have different ECM components, and specific ECM compositions can enhance the migration and proliferation of specific cells (Karamanos et al., 2021). The pathological changes associated with psoriasis mainly reflect massive proliferation of keratinocytes in the epidermis, an increase in the number of dead cells in the surface layer, and the migration of keratinocytes to the dermis (Kellner et al., 1991; McFadden et al., 2012). Alterating the ECM is one of the main ways that keratinocytes break out of their intrinsic topology and migrate towards the dermis (Kellner et al., 1991; Ghaffari et al., 2006; Philips et al., 2009).

The ECM is composed of an intricate blend of structural and functional macromolecules, and it contributes to the morphogenesis of tissues and organs and sustains the architecture and function of cells and tissues (Wagner et al., 2021). Cell–ECM interactions are facilitated by specific transmembrane molecules, primarily integrins, and possibly by other cell surface-associated components, such as proteoglycans, CD36, and similar entities (Glinski et al., 1995; McFadden et al., 2012). These interactions exert direct and indirect effects through various cellular activities, such as adhesion, migration, differentiation, proliferation, and apoptosis (Glinski et al., 1995; McFadden et al., 2012; Wagner et al., 2021). Moreover, integrins serve as mechanoreceptors, establishing a physical connection that enables force transmission between the ECM and the cytoskeleton (Wagner et al., 2021).

The ECM plays a crucial role in maintaining tissue homeostasis and facilitating repair across all organs. In the past, it was predominantly seen as being inactive and merely providing structural support for the tissue (Karamanos et al., 2021). However, through detailed examinations of the physical and biochemical attributes of the ECM, a wide range of functions have been revealed, enhancing our previous understanding. Today, it is evident that the ECM is a key component of tissues that supports and sustains dynamic interactions between different cellular compartments and resident cells. The ECM can also recruit inflammatory cells (TH1, TH2, TH17, TH22) and proinflammation proteins (IL-6, IL-8, IL-17 and HLA-1,2) in response to pathological stimuli (McFadden et al., 2012; Pfisterer et al., 2021; Wagner et al., 2021; Clément et al., 2022; Schett et al., 2022). Additionally, mutations in ECM proteins have the potential to result in a range of genetic conditions. For examples, Genome-wide studies associate LAMA5 mutations with psoriasis susceptibility. Psoriatic lesions show elevated fibronectin, driven by TGF-β and IL-22. FN1 variants correlate with disease severity. Type I/III collagen degradation by MMP-9 which is upregulated in psoriasis promotes epidermal thickening. Rare COL6A5 mutations are linked to early-onset psoriasis. Researchers have investigated the composition, structure, and function of the ECM in the context of skin homeostasis and inflammatory-based skin diseases such as psoriasis, offering a model for understanding the broader impact of the ECM on human health (Holm Nielsen et al., 2021; Pfisterer et al., 2021; Wagner et al., 2021).

## 2 miRNA-mediated regulation of the cytoskeleton in psoriasis

### 2.1 miRNAs regulate the cytoskeleton

miRNAs are compact noncoding RNA molecules that are 21–24 nucleotides (nt) in length and are capable of controlling the expression of genes at the posttranscriptional level by targeting coding and noncoding RNAs within competing endogenous RNA (ceRNA) networks (O’Brien et al., 2018). Over 250 miRNAs have been shown to play crucial roles in the regulation of skin inflammation and the maintenance of skin homeostasis (Hawkes et al., 2016; Jiang et al., 2023). In psoriasis, miRNAs shown different expression patterns in different cell types or at different sites (Guinea-Viniegra et al., 2014; Wcisło-Dziadecka et al., 2018; El-Komy et al., 2020; Xiao et al., 2020; Solvin et al., 2022; Ganguly et al., 2024).

The abnormal regulation of miRNA transcription has been implicated in different aspects of psoriasis pathogenesis, including cytoskeletal remodeling. Several miRNAs, namely, miR-21, miR-146a, miR-146b and miR-203, exhibit increased expression in keratinocytes, fibroblasts and the blood, and they modulate cytoskeletal dynamics by targeting genes involved in actin filament organization, microtubule stability, and intermediate filament formation (Chatzikyriakidou et al., 2010; McKenna et al., 2010; Hermann et al., 2017; Xiao et al., 2020; Henriet et al., 2023). Other miRNAs, such as miR-4516, miR187, miR-876, miR-183, miR-181, miR-194, and miR-124, are downregulated and regulate cytoskeletal changes associated with the cell cycle in psoriatic keratinocytes to accelerate migration, alter adhesion, and promote resistance to apoptosis (Song et al., 2013; Chowdhari and Saini, 2014; Chowdhari and Saini, 2016; Chakraborty et al., 2016; A et al., 2018; Tang et al., 2019; Zhang et al., 2023). Understanding the specific miRNA‒mRNA interactions that regulate cytoskeleton-related genes can provide meaningful insights into the mechanisms underlying cytoskeletal abnormalities in psoriasis.

miRNAs regulate the cytoskeleton in psoriasis by targeting key genes involved in these processes. For example, miR-21 has been shown to target RhoB, a small guanosine triphosphate involved in actin dynamics, leading to increased actin polymerization and cellular migration in psoriatic keratinocytes (Sabatel et al., 2011; Hessam et al., 2017; Chandan et al., 2020). miR-203 has been found to downregulate the expression of p63, a transcription factor important for epidermal differentiation and keratinocyte adhesion. This dysregulation disrupts the normal formation of adherens junctions and contributes to pathological changes in the psoriatic epidermis (McKenna et al., 2010; Liu et al., 2012; Hessam et al., 2016; Chen et al., 2018; Chen et al., 2021). Additionally, miR-146a targets interleukin-17 receptor C (IL17RC), a key receptor involved in the inflammatory response in psoriasis, leading to the modulation of keratinocyte proliferation and migration (Chatzikyriakidou et al., 2010; Joyce et al., 2011; Hermann et al., 2017; Chen et al., 2018; Wei et al., 2022).

The metabolic rates of psoriatic keratinocytes exceed those of keratinocytes in healthy skin (Jiang et al., 2019; Liu et al., 2019; Takano et al., 2020). This change results in abnormal protein folding, leading to a loss of protein function. Consequently, hyperproliferating keratinocytes require an increased amount of heat shock proteins (HSPs) to ensure the correct folding of substrate proteins in signaling pathways. F-actin polymerization and actin cytoskeleton organization are influenced by HSPB1. The dysregulation of miRNA-22 can cause low expression of HSPB1. The downregulation of miRNA-22 was observed in lesions from human psoriatic skin [23, 50, 74]. In addition, HSPB1 phosphorylation is suppressed when human homolog of the *drosophila* tumor suppressor l (hTid-1S) binds to map kinase-activated protein kinase 5 (MK5). This change decreases the polymerization of F-actin. The loss of hTid-1S expression is related to abnormal actin organization in psoriasis (Choi et al., 2012).

Moreover, the rearrangement of F-actin induced by activated p38 mitogen-activated protein kinases (p38MAPK) is predominantly facilitated by MK2, which phosphorylates HSPB1. At least three proteins are involved in the abnormal expression of components of the p38MAPK signaling pathway in psoriasis. p38MAPK has been shown to play an important role in the polymerization of F-actin, actin filament dynamics and actin cytoskeleton organization in different types of cells (Kim et al., 2020; Bai et al., 2023; Zhang et al., 2023). Furthermore, miR-21, miR-125, miR-148, miR-199a-3p, and miR-126 regulate the NF-KB1, MAPK and SERPINB4 proteins in psoriasis (Zhang et al., 2023). Interestingly, active miRNAs in psoriasis target MAP kinases; for example, miRNA-148 transcriptionally regulates MAPK1, MAP2K3, MAP3K4, and MAP4K3 (Helwak et al., 2013; Pelosi et al., 2018).

### 2.2 Regulation of the cytoskeleton and ECM via the miRNA-related network

The miRNA-related regulatory network is a complex system that involves the interaction of miRNAs with mRNAs, lncRNAs, circRNAs, pseudogenes and other molecules to control gene expression; this network involves miRNA‒protein, mRNA‒miRNA–protein, lncRNA‒miRNA–protein, circRNA–miRNA‒protein, and pseudogene–miRNA‒protein interactions (Li et al., 2014; Tay et al., 2014; Jin et al., 2020).

The protein CCHCR1, which is located in prominent psoriasis susceptibility region PSORS1, is a strong candidate gene linked to the psoriasis risk allele CCHCR1* HLA-Cw6 (Tervaniemi et al., 2012; Tervaniemi et al., 2018). It exhibits altered expression in psoriasis lesions compared to normal skin. Its overexpression affects the proliferation of mouse cells. Cells overexpressing CCHCR1 exhibit isoform- and haplotype-specific changes in the area and shape of the cells, leading to changes in the organization and expression of cytoskeletal proteins such as actin, vimentin, and cytokeratin (Tervaniemi et al., 2012; Tervaniemi et al., 2018). The localization of CCHCR1 to the centrosome establishes a possible connection to aberrant cell proliferation and offers insight into the cellular pathways that may be altered in psoriasis. These discoveries shed light on the intricate role of CCHCR1 in psoriasis development, underscoring the need for further investigations of its potential as a therapeutic target (Tervaniemi et al., 2012; Tervaniemi et al., 2018). Interestingly, the changes in CCHCR1 expression observed in keratinocytes from psoriatic lesions involve the interaction of miRNAs and endogenous competing RNAs (ceRNAs). Following experiments involving interleukin 22 (IL22) treatment in different types of psoriatic cells, there was a marked accumulation of MSX2P1. Song et al. (2021) investigated a ceRNA network called MSX2P1-miR-6731-5p-S100A7. They discovered a positive association between the production of MSX2P1 and S100A7. Through luciferase reporter assays, they showed that miR-6731-5p was directly targeted by MSX2P1, resulting in the downregulation of miR-6731-5p expression. This downregulation promoted cell proliferation; suppressed apoptosis stimulated by IL-22 in keratinocytes; and increased the expression of various proinflammatory cytokines, including S100A7, IL12B, IL23, TNF-α, human leukocyte antigen (HLA-C), coiled-coil alpha-helical rod protein 1 (CCHCR1), and nuclear factor proteins (Song et al., 2021). These findings suggest that lncRNA-MSX2P1 can bind to miR-6731-5p and function as a sponge RNA. As a result, the inhibition of miR-6731-5p expression occurs, leading to increases in the levels of S100A7 and other inflammatory cytokines in keratinocytes stimulated with IL22. This mechanism contributes to the onset of psoriasis. miR-6731-5p was also found to impair cell proliferation, increase apoptosis in keratinocytes stimulated with IL-22, and decrease the levels of S100A7, IL12B, IL23, HLA-C, CCHCR1, TNF-α, and NF-κB. MSX2P1 enhances the proliferation of IL22-secreting keratinocytes by suppressing miR-6731-5p and stimulating S100A7 (Qiao et al., 2018). Therefore, targeting the mRNA‒miRNA‒lncRNA network MSX2P1‒miR-6731‒5p‒S100A7 could be a promising and innovative approach for treating psoriasis in the future (Qiao et al., 2018; Song et al., 2021).

LncRNA H19 harbours two binding sites for miRNA-130b-3p (Song et al., 2021). When miRNA-130b-3p was introduced into cells, the activity of wild-type H19 markedly decreased, and the effect on mutant H19 was less pronounced. Elevated miRNA-130b-3p levels in keratinocytes led to a reduction in the expression of H19. Throughout keratinocyte differentiation, H19 expression increases, accompanied by a decrease in miR130b-3p levels. Knocking down H19 results in reduced expression of DSG1 and subsequently leads to decreased keratinocyte numbers after calcium stimulation (Zibert et al., 2010; Chandra et al., 2015; Song et al., 2021). Moreover, H19 counteracts the inhibitory effects of DSG1 expression associated with miR-130b-3p. In addition, a protective agent targeting DSG1 alleviated the inhibitory effect of H19 knockdown on keratinocyte differentiation (Song et al., 2021). These findings suggest that DSG1-miR-130b-3p-lncRNA-H19 could represent a promising novel target for treating psoriasis.

In fact, although numerous studies have identified genes associated with cytoskeletal and ECM alterations in psoriasis, the regulatory miRNAs controlling these genes remain largely uncharacterized. To address this knowledge gap, a bioinformatics-driven approach is essential. Adhesion G protein-coupled receptor F4 (ADGRF4) has been reported to be involved in the development of inflammatory skin diseases, including psoriasis. It is also involved in the response to glucocorticoid therapy in patients with these illnesses (Wang et al., 2018). Winkler et al. (2022) reported promising results related to the localization of ADGRF4 inside the nucleus of keratinocytes in both psoriasis skin samples and cultured psoriatic cells. The ADGRF4 protein can be detected in virtually all specialized suprabasal and velum keratinocytes in normal skin. In contrast, ADGRF4 expression is decreased in psoriatic skin exhibiting excessive proliferation and irregular differentiation of keratinocytes. The detection of ADGRF4 in MKI67-positive suprabasal keratinocytes suggests that ADGRF4 is involved in epidermal differentiation. Moreover, the deletion of ADGRF4 using CRISPR/Cas9 resulted in a reduction in the number of keratinocyte layers and elimination of keratin 1 (KRT1) expression. Another study revealed that disruption of pituitary tumour transforming gene 1 (PTTG1) expression led to a decrease in epidermal stratification, causing the cells to exhibit a tendency to appear as a simple epithelium (Wang et al., 2012; Winkler et al., 2022). The levels of both lymphoid-specific helicase (HELLS) and RING-finger type E3 ubiquitin ligases 1 (UHRF1) are reduced in a thinner epidermis with disrupted epidermal homeostasis (Wang et al., 2012). In light of the findings described above, ADGRF4 may direct KRT1 to regulate epidermal differentiation during the early stage of the disease; moreover, it is expressed in a small number of proliferating basal cells (Wang et al., 2012). The specific miRNAs related to ADGRF4, KRT1, HELLS, and UHRF1 in psoriasis have not been reported in the literature (Joyce et al., 2011; Pelosi et al., 2018; Singhvi et al., 2018). However, in bioinformatics prediction databases such as TargetScan, each of these proteins has more than 100 related miRNAs that can bind to the mRNAs that are transcribed to generate these proteins. miRNAs promote mRNA degradation to block their expression. Clustering and module analysis of miRNAs is important for identifying miRNAs that may effectively regulate important functionally altered proteins in biological experiments. These findings can lead to reliable bioinformatics analyses of potential biomarkers as well as therapeutic drugs. This could enable researchers to better understand and analyze the collaboration and competition that occur in the context of psoriasis at multiple levels.

In addition, researchers have recently identified epidermal keratin near peripheral endoplasmic reticulum (ER) compartments at desmosomes. These keratins play a vital role in preserving the quality of desmosome-associated ER compartments. This was demonstrated by a study showing that the expression of a disease-associated KRT14 mutation resulted in the disorganization of endoplasmic reticulum–desmosome complexes (Bharathan et al., 2022).

Overall, the miRNA-mediated regulation of the cytoskeleton and ECM in psoriatic tissues is very complicated. In addition to miRNA clustering and the miRNA‒mRNA regulation mode, the lncRNA‒miRNA‒mRNA regulatory ceRNA network is beneficial for screening representative miRNAs that strongly affect the cytoskeleton and ECM.

## 3 Exosomes, the cytoskeleton and ECM regulation in psoriasis

### 3.1 Exosomal miRNAs affect the cytoskeleton and ECM in psoriasis

**FIGURE 2:**
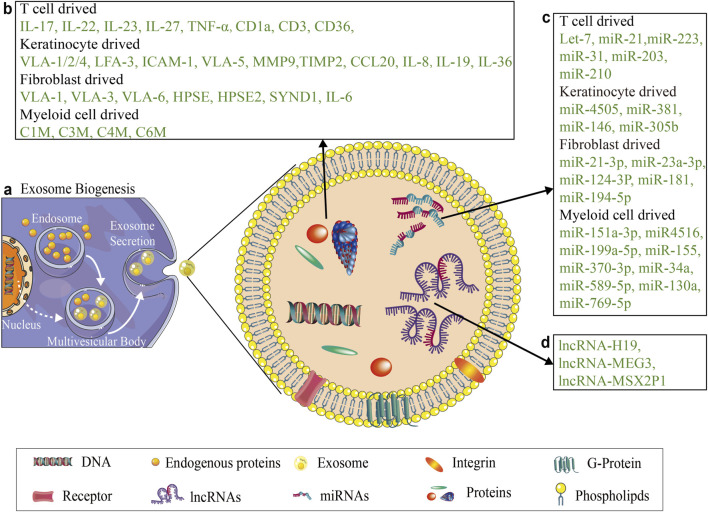
Main proteins and ncRNAs in exosomes secreted by different cells in the context of psoriasis. **(a)** Shows the simple biogenesis of exosome. **(b)** Illustrates the major protein components of exosomes secreted by key cellular populations in psoriasis, including immunomodulatory somatic cells, T cells and Myeloid cells, as well as structural cells, keratinocytes and fibroblasts. **(c)** Shows the major miRNA of exosomes secreted by key cellular populations in psoriasis, including T cells and Myeloid cells, keratinocytes and fibroblasts. **(d)** Illustrates the lncRNA encapsulated within exosomes isolated from psoriasis patients.

Exosomes are nanosized vesicles secreted by cells that mediate intercellular communication by transferring biomolecules like miRNAs and proteins (Théry et al., 2002). In psoriasis, exosomes facilitate crosstalk among Myeloid cells, T cells, fibroblasts and keratinocytes, serving as both disease biomarkers and potential therapeutic vehicles (Cheung et al., 2016; Clément et al., 2022; Hussain et al., 2022; Mastronikolis et al., 2023; Dehghani et al., 2024; Iuliano et al., 2024) (see Figure 2).

Exosomes are essential for intercellular communication because they transport their cargo, including miRNAs, to recipient cells. In the context of psoriasis, exosomes can be isolated from activated immune cells, including dendritic cells and T cells, and can transfer miRNAs that modulate cytoskeletal dynamics and ECM remodeling in keratinocytes and fibroblasts (Clément et al., 2022; Iuliano et al., 2024). For example, miR-155, which is enriched in exosomes from activated T cells, has been reported to target genes involved in cytoskeletal organization and adhesion, leading to altered migration and invasion of psoriatic keratinocytes (García–Rodríguez et al., 2017; Zhang et al., 2022). Moreover, exosomal miR-223, which is released by activated immune cells, has been shown to regulate matrix metalloproteinases (MMPs) and tissue inhibitors of metalloproteinases (TIMPs), influencing ECM remodeling and tissue homeostasis in psoriasis (Tse, 2003; Hessam et al., 2017; Born and Khachemoune, 2023). Furthermore, exosomal miRNAs, such as miR-203, miR-4505, and miR-23a, play a role in targeting ECM components, MMPs, and TIMPs, thus impacting ECM remodeling and integrity (Tse, 2003; Delić et al., 2020; Sinkeviciute et al., 2020; Wagner et al., 2021; Mastronikolis et al., 2023; Sun et al., 2023). Furthermore, exosomes isolated from psoriatic cells have been shown to activate proinflammatory responses and facilitate abnormal ECM deposition (Jiang et al., 2019; Paolino et al., 2022; Zhang et al., 2022).

**FIGURE 3:**
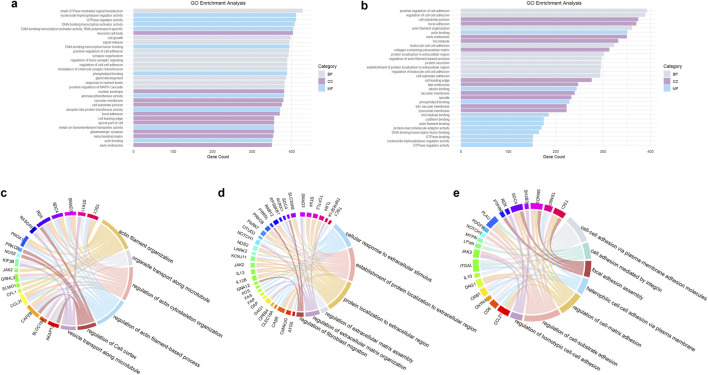
GO enrichment analysis. **(a)** Shows the GO analysis of TargetScan- and miRbase-predicted target genes of differentially expressed exosomal miRNAs in psoriasis (top 30). **(b)** Shows the main enriched GO terms related to the cytoskeleton, ECM and focal adhesion based on the same miRNAs shown in a (top 30). Chord diagrams show the relationships between genes and biological processes based on an intersection of the data from TargetScan and miRanda. **(c)** Shows the cytoskeleton-related genes and their biological processes; **(d)** Shows ECM-related genes and their biological processes; and **(e)** is the adhesion-associated genes and their biological processes. GO analysis shown here is derived from aggregated published data on psoriasis-associated miRNAs (McKenna et al., 2010; Mezentsev et al., 2014; Hermann et al., 2017; Hessam et al., 2017; Chen et al., 2018; Chen et al., 2019; Magenta et al., 2019; Lv et al., 2020; Martínez-Hernández et al., 2020; Xiao et al., 2020; Bychkov et al., 2022; Sun et al., 2023; Iuliano et al., 2024; Yu et al., 2024).

Exosomal miRNAs from psoriatic skin lesions and peripheral blood samples were comparatively profiled, revealing shared miRNAs implicated in the regulation of cytoskeletal organization and ECM modulation. These miRNAs include miR-21-3p, miR-378a-3p, miR-146a, miR-200c, miR-223, miR-143, miR-31, miR-124, miR-203, hsa-miR-381-3p, hsa-miR-486-5p, miR-30e-5p, let-7d-5p, miR-151a-3p, miR-199a-5p, miR-370-3p, hsa-miR-589-5p, hsa-miR-769-5p, hsa-miR-21, hsa-miR-155, miR-23a-3p, miR-1305, miR-381-3p, and hsa-miR-4505 (McKenna et al., 2010; Mezentsev et al., 2014; Hermann et al., 2017; Hessam et al., 2017; Chen et al., 2018; Chen et al., 2019; Magenta et al., 2019; Lv et al., 2020; Martínez-Hernández et al., 2020; Xiao et al., 2020; Bychkov et al., 2022; Sun et al., 2023; Iuliano et al., 2024; Yu et al., 2024). Many of these miRNAs influence actin filament organization by targeting genes that regulate actin polymerization and depolymerization, affecting cell shape, motility, and stability. miRNAs such as miR-200c, miR-31, and miR-203 target genes involved in cell‒cell adhesion, such as E-cadherin, preserving epithelial integrity and preventing excessive cell migration (Magenta et al., 2019; Shao et al., 2020; Xiao et al., 2020). These miRNAs regulate the expression of MMPs, TIMPs, collagen, and other ECM components, ensuring controlled ECM turnover and preventing fibrosis or excessive matrix degradation (Ghaffari et al., 2006; Iuliano et al., 2024; Qi and Jin, 2024). miR-21-3p is known to influence actin cytoskeleton dynamics by targeting proteins involved in cytoskeletal remodeling. It can also stimulate the formation of MMPs and other ECM-related proteins, affecting the composition and degradation of the ECM (Degueurce et al., 2016). miR-378a-3p can modulate genes involved in cytoskeletal organization, such as those encoding actin-binding proteins (Soonthornchai et al., 2021). miR-146a can regulate genes involved in cell adhesion and cytoskeletal dynamics, influencing cell shape and motility (Chatzikyriakidou et al., 2010). miR-200c inhibits EMT by targeting ZEB1/2, maintaining epithelial characteristics and cytoskeletal stability. miR-200c regulates MMPs and TIMPs, ensuring balanced ECM remodeling. miR-223 modulates actin cytoskeleton dynamics by targeting proteins involved in actin polymerization and depolymerization (Hessam et al., 2017; Srivastava et al., 2021). miR-143 targets genes involved in cytoskeletal organization, affecting cell shape and motility (Glinski et al., 1995; Zeng et al., 2016). miR-31 modulates genes involved in cytoskeletal dynamics and cell adhesion, influencing cell movement and stability. It can also affect the ECM composition by controlling the expression of MMPs and other ECM-related proteins (Liu et al., 2019; Born and Khachemoune, 2023). miR-203 targets genes involved in maintaining epithelial cell adhesion and cytoskeletal stability (Chen et al., 2018; Xiao et al., 2020). TargetScan (McGeary et al., 2019) and miRbase (Kozomara et al., 2019) have been utilized to predict the target genes of the exosomal miRNAs mentioned above. R 4.3.2 (R Core Team, 2023) has also been utilized for GO enrichment analysis. These studies proved that these exosomal miRNAs play roles in changes in the cytoskeleton and ECM (see Figures 3a,b). The target genes or proteins of these miRNAs were enriched in biological processes involved in establishing the cell structure and microenvironment, including “cell growth,” “signal release,” “positive regulation of cell adhesion,” “regulation of cell-cell adhesion,” and “positive regulation of MAPK cascade” (see Figure 3a), as well as “actin filament organization,” “protein localization to extracellular region,” and “cell-substrate adhesion” (see Figure 3b). The MAPK pathway is strongly related to cell differentiation, proliferation and apoptosis. Regarding cellular components in the enrichment analysis, “vacuolar membrane,” “cell-substrate junction,” “focal adhesion,” “cell leading edge,” “apical part of cell,” and “early and late endosome” were found to be enriched, as shown in both Figures 3a,b, whereas “microtubule,” “collagen-containing extracellular matrix,” “lytic vacuole membrane” and “lysosomal membrane” are only shown in Figure 3b. Molecular functions such as actin binding and microtubule binding are shown in Figures 3a,b. Potential proteins and genes involved in psoriatic cytoskeleton-related, ECM-related and adhesion-related biological processes are shown in Figures 3c–e, respectively.

### 3.2 Exosomal proteins involved in psoriasis affect the cytoskeleton and ECM

Exosomes contain a wide array of proteins, ranging from structural proteins to proteins involved in the immune response. These proteins include tetraspanins, HSPs, endosomal sorting complex required for transport proteins, cytoskeletal proteins, membrane fusion and transport proteins, signal transduction proteins, integrins and adhesion molecules (Gurung et al., 2021; Lai et al., 2022). Exosomal proteins play crucial roles in the regulation of the cytoskeleton and ECM, impacting cellular processes that play important roles in the pathology of psoriasis (Wang et al., 2018; Iuliano et al., 2024).

Higher levels of tetraspanins, such as CD1 and CD3, are found in exosomes from psoriatic patients, indicating enhanced exosome release, altered communication between keratinocytes and immune cells, and changes in the ECM in different cell types. In psoriasis patients, Langerhans cells (LCs) exhibit impaired migration, contributing to the pathogenesis of the disease. LCs from the epidermis constitutively express CD1a (Cheung et al., 2016). Previous studies have reported increased numbers of mast cells in psoriatic lesions. This evidence strongly suggests that T cells are the most important cell type in psoriatic development. Recent findings suggest that ECM proteins (collagens and fibronectin) play a costimulatory role in the lymphoproliferative responses of T cells *in vitro*. Researchers have investigated protein costimulatory activities in psoriatic individuals. They demonstrated that T cells that penetrate the skin experience uninterrupted exposure to collagen and fibronectin. On the one hand, the proliferative response of CD3-activated T cells is reduced in psoriasis; on the other hand, this process can be augmented by collagen I and collagen IV, as well as fibronectin (McFadden et al., 2012). Notably, costimulation via collagen I substantially decreased in psoriasis patients, whereas an increase was observed after treatment with collagen IV, indicating an improved response. These alterations were more regularly observed in patients with active and widespread lesions. These findings indicate that abnormalities in signals derived from ECM proteins could contribute to the immunopathology of psoriasis. Exosomes seem to establish a communication “loop” in this process (Holm Nielsen et al., 2023).

Moreover, Glinski et al. (1995) examined the costimulation of proliferative T-cell responses triggered by anti-CD3 using collagen I and IV. The stimulation rate in response to anti-CD3 was reduced by approximately 50% after exposure to collagen I (Glinski et al., 1995). Notably, in individuals with widespread, active plaque psoriasis, the proliferative response of CD3^+^ lymphocytes decreased by approximately half. However, costimulatory responses associated with collagen IV and fibronectin have been reported to be heightened compared with those in healthy individuals. These results suggest that the number of T cells carrying receptors for specific ECM proteins that are carried by exosomes is increased in the peripheral blood, potentially due to the migration of T cells through the basement membrane zone of lesions in widespread, active plaque psoriasis. This process may lead to altered responsiveness of T cells following spreading and scattering (Glinski et al., 1995).

In addition, Cheung et al. (2016) reported that the number of mast cells releasing exosomes can be increased by IFNA. Circulating CD1a-autoreactive T cells are more abundant in psoriatic individuals and react to PLA2-exosomes from the LAD2 mast cell–like line, leading to increased secretion of IL22 and IL17 in T cells. These results suggest that neolipids are of considerable importance in activating T cells, with exosomes serving as essential carriers for neolipids. While these results suggested that targeting PLA2 or CD1a holds promise as a possible new therapy for psoriasis, further research is necessary to confirm this possibility (Cheung et al., 2016).

Exosomes from psoriasis patients contain high levels of proinflammatory cytokines and chemokines, which are partly responsible for the inflammatory milieu that is characteristic of psoriatic lesions. Both HPSE and HPSE2 exhibited considerably increased protein and mRNA expression in psoriatic lesions compared with non-affected skin. In psoriasis plaques, the levels of MMP9 and TIMP2 were greater than those in skin from healthy controls. There is a potential correlation between the distinctive inflammatory changes associated with psoriasis and changes in the ECM. The increased expression of HPSE2, SYND1, MMP9, and TIMP2 observed in psoriasis patients, even when psoriatic plaques are not present, suggests their involvement in primary changes associated with psoriasis and their potential as candidate proteins for targeted treatments to reverse ECM modifications (Wagner et al., 2021). MMP levels are altered in exosomes from the epidermis of psoriasis patients. These proteins can alter intracellular communication and change to the composition of the ECM, facilitating angiogenesis in the dermal vasculature and immune cell infiltration (Mezentsev et al., 2014).

Proteases and other ECM-modulating proteins in exosomes can change and remodel the ECM, facilitating the invasion of immune cells into the skin and perpetuating inflammation. Type VI collagen, which contains the collagen VI α6 chain, is upregulated in exosomes from atopic dermatitis and psoriasis patients (Holm Nielsen et al., 2023).

Exosomes carry integrins and other adhesion molecules that can influence cell‒matrix interactions, affecting cell adhesion, migration, and invasion. This phenomenon is particularly important in psoriasis, where altered cell adhesion and increased keratinocyte migration contribute to plaque formation. A study revealed that after blocking the interaction between α1β1 integrin (ITGA1) and collagen, epidermal T cells accumulate and prevent psoriasis immunopathology. Notably, ITGA1, an important collagen-binding surface receptor, was exclusively expressed in epidermal T cells but not T cells in the dermis. T cells with very high expression of ITGA1 exhibited surface markers typical of effector memory cells and elevated levels of interferon-c, whereas IL4 was not present. These findings emphasize the crucial role of ITGA-1 in regulating the accumulation of type 1-polarized effector memory T cells in the epidermis, a characteristic shared by psoriasis and similar immunopathologies (Conrad et al., 2007). Compared with those in healthy controls, the intensity and localization of the adhesion molecules ITGA1, ITGA2, ITGA4, and LFA-3 in psoriatic lesions was not altered. In contrast, ITGA3 and ITGA6 are normally limited to basal keratinocytes in healthy skin and were found to be present in abnormally high numbers among spinous cells from psoriatic lesions. Additionally, in normal skin, keratinocytes do not express ICAM-1 and ITGA-5, which show focal induction, particularly in cells located above elongated rete ridges and in areas with intraepidermal granulocyte and lymphocyte infiltration (Kellner et al., 1991).

Exosomal proteins activate signaling pathways in recipient cells, resulting in alterations in gene expression and cellular behavior that promote the psoriatic phenotype. Exosomes carry proinflammatory cytokines such as TNF-α and IL1B, which exacerbate the inflammatory environment in psoriasis, leading to increased keratinocyte proliferation and altered ECM composition. Psoriasis may lead to psoriatic arthritis (PsA), which is linked to progressive joint damage and substantial physical impairment in patients. Exosomes obtained from blood samples from PsA patients have been shown to increase osteoclastogenesis (Chen et al., 2019). Interestingly, this stimulatory effect on osteoclastogenesis was not associated with age, disease duration, disease severity, C-reactive protein levels, or rates of erythrocyte sedimentation. This discovery implies that exosomes may serve as potent and context-dependent regulators of osteoclastogenesis in humans.

The role of exosomes in the production of cellular junctions such as focal adhesions and ECM components such as collagen and connexin provides new ideas for the treatment of psoriasis. The structure of exosomes has inspired the study of drug carriers at the nanoscale. The interaction of proteins contained in exosomes with the cytoskeleton and ECM has elucidated the specific alterations in adhesion molecule expression in psoriasis, potentially contributing to the understanding of psoriasis pathophysiology and laying the foundation for the exploration of new psoriasis treatment strategies centered on targeting the ECM interactions of T cell–keratinocyte and T cell–fibroblast pairs. Continuous ECM remodeling in the skin is essential for maintaining tissue homeostasis.

## 4 Crosstalk among miRNAs, exosomes, the cytoskeleton, and the ECM in psoriasis

**FIGURE 4:**
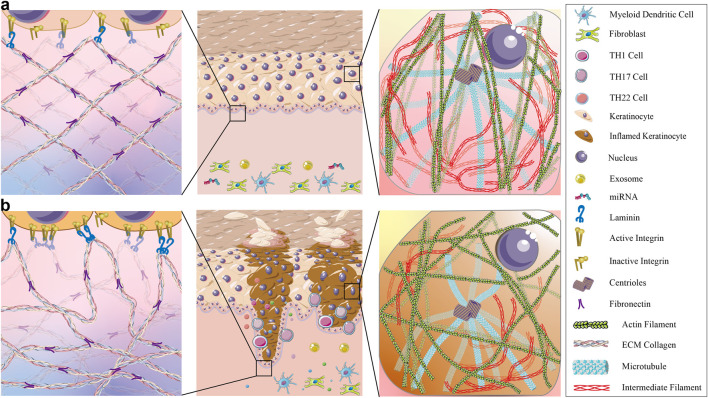
The differences in ECM and keratinocyte cytoskeleton between normal skin and psoriatic skin. **(a)** Shows that in healthy skin, the extracellular matrix (ECM) is well-organized, forming a stable spatial structure with densely packed basal keratinocytes. This ensures robust signal transduction between the epidermal and dermal layers. The keratinocyte cytoskeleton is evenly distributed: microtubules mediate precise vesicle trafficking, microfilaments bundle to reinforce cell adhesion and division, and intermediate filaments maintain cellular morphology and enhance mechanical strength. **(b)** shows that in psoriatic lesional skin, excessive inflammatory cytokines like IL-6, IL-17, IL-22 and hyperactivate integrins, disrupting laminin distribution and resulting in a fragmented and disorganized ECM. Fibronectin forms dense, irregular aggregates. The keratinocyte cytoskeleton exhibits severe abnormalities—microtubule hyperpolarization leads to aberrant vesicle accumulation, disorganized and truncated microfilaments impair cell adhesion while accelerating proliferation, and reduced intermediate filaments destabilize cellular architecture.

The interplay among miRNAs, exosomes, cytoskeleton dynamics, and ECM remodeling in psoriasis is highly complex (see Figure 4). miRNAs can regulate the expression of exosomal cargo, whereas exosomal miRNAs can modulate the activity of target genes involved in cytoskeleton organization and ECM remodeling. Additionally, altered cytoskeletal dynamics can impact exosome biogenesis and release, influencing their cargo composition and function (Théry et al., 2002; Hessvik and Llorente, 2018). This intricate network of interactions is involved in the pathogenesis of psoriasis and offers potential therapeutic targets for intervention.

## 5 Influence of treatments on miRNAs and exosomes

### 5.1 Typical treatment

Psoriasis is generally categorized into five types (Armstrong and Read, 2020; Lee and Kim, 2023). Plaque psoriasis, the most prevalent type, appears as raised, red patches with a silvery scaly surface. Guttate psoriasis is characterized by small, pinpoint lesions and is frequently induced by infections. Inverse psoriasis is characterized by shiny, red lesions in the skin folds, whereas pustular psoriasis is characterized by white pustules encircled by red, inflamed skin. Erythrodermic psoriasis, the most severe form, causes widespread redness and severe itching. Nail psoriasis affects the nails through pitting and discolouration, and PsA involves joint pain and swelling, often resembling rheumatoid arthritis (Talotta et al., 2019).

There are currently many treatments for psoriasis, including chemicals, biologics, traditional Chinese medicines, phototherapy, and emerging therapies, including microneedles and nanocarriers (Armstrong and Read, 2020; Mohd Nordin et al., 2021; Lv et al., 2022; Lee and Kim, 2023). Clinical treatment can be selected on the basis of the severity of the disease. Mild psoriasis typically affects no more than 3%–5% of the skin area. Topical corticosteroids, vitamin D analogues, calcineurin inhibitors, keratolytics, and targeted phototherapy are used to treat mild psoriasis on the basis of the location of the lesions, the existence of comorbidities, and the patient’s personal preferences (Nylander et al., 2011; Kocic et al., 2019; Holm Nielsen et al., 2023; Lee and Kim, 2023; Sun et al., 2023; Iuliano et al., 2024). Moderate psoriasis affects 5%–10% of the skin surface area, whereas severe psoriasis affects more than 10% of the body surface area. Systemic treatments are considered the cornerstone in the management of moderate-to-severe psoriasis, and these treatments can also be effective in patients with localized disease for whom topical therapies have proven inadequate (Armstrong and Read, 2020; Reid and Griffiths, 2020). Systemic treatment involves a combination of biologics, oral agents, and phototherapy (Lowes et al., 2007; Armstrong and Read, 2020; Reid and Griffiths, 2020). Biologics have demonstrated greater efficacy than oral medications and phototherapy (Rapalli et al., 2018; Rapalli et al., 2020; Lee and Kim, 2023). While topical therapies can serve as supplementary treatments, they are insufficient as standalone therapies for moderate-to-severe psoriasis (Rapalli et al., 2020; Reid and Griffiths, 2020).

Different treatments affect the release of exosomes and miRNAs in psoriatic lesions and the blood. These changes can reveal differentially expressed miRNAs and exosome alterations in psoriasis, providing a basis for bioinformatics research and the identification of biomarkers.

### 5.2 Changes in miRNA expression levels in different treatment groups

miRNAs are basic regulators of the pathogenesis of psoriasis, influencing various aspects of the disease, including inflammation, cell proliferation, and cytokine responses (Huang et al., 2015; Jiang et al., 2023). Changes in miRNA expression levels are associated with disease severity and treatment response in psoriasis patients (Abdallah et al., 2023; Ganguly et al., 2024). For example, the levels of miR-135b were significantly reduced following treatment with methotrexate, though no such effect was observed after NB-UVB therapy (Joyce et al., 2011; Aydin et al., 2020). A similar decrease in miR-135b expression was noted in patients receiving biologic therapies targeting inflammatory pathways, including anti-IL-12/23, anti-TNFα, and anti-IL-17 (Chicharro et al., 2020). This suppression across treatments suggests miR-135b downregulation is a shared therapeutic outcome mediated through distinct mechanisms—methotrexate potentially via adenosine-mediated immunosuppression or STAT3 inhibition, anti-IL-12/23 and anti-IL-17 biologics through IL-23/Th17 axis disruption, and anti-TNFα agents via NF-κB modulation (Aydin et al., 2020; Xiao et al., 2020). Notably, miR-135b, like miR-203, regulates keratinocyte proliferation in the basal layer of epidermis, with both miRNAs implicated in maintaining epidermal homeostasis (Joyce et al., 2011; Aydin et al., 2020). Their dysregulation in psoriasis exacerbates hyperproliferation and disrupts tight junction integrity, further amplifying inflammation. The correlation between reduced miR-135b/miR-203 levels, improved the psoriasis area and severity index scores, and normalized keratinocyte organization underscores their dual role in linking aberrant proliferation and inflammatory signaling (Aydin et al., 2020). Similarly, the levels of specific miRNAs, such as miR-147b, miR-3614-5p, and miR-125a-5p, differed considerably between patients with mild and severe psoriasis (Ganguly et al., 2024). Studies have also evaluated the roles of various specific miRNAs in psoriasis pathogenesis. For example, miR-145-5p expression was found to be downregulated in psoriasis, contributing to hyperproliferation of keratinocytes and skin inflammation (Yan et al., 2019).

Additionally, the expression of miRNAs such as miR-155, miR-210, and miR-20b is upregulated in psoriasis patients, with miR-155 specifically linked to disease activity and IL17 production (El-Komy et al., 2020; Garcia-Martin et al., 2022). Furthermore, the expression of miRNAs such as miR-223 and miR-143 is downregulated in peripheral blood mononuclear cells (PBMCs) following treatment with methotrexate, which coincides with decreased psoriasis severity (Huang et al., 2015). The dysregulation of miRNAs in psoriasis can impact various cellular processes. For example, miRNAs such as miR-203 have been shown to modulate signaling pathways that contribute to psoriasis progression, such as the JAK2/STAT3 pathway (Shen et al., 2022).

Moreover, miRNAs can regulate immune responses in psoriasis, with miR-155 being implicated in promoting inflammatory processes by increasing IL17 production (El-Komy et al., 2020). Additionally, miRNAs have been suggested as promising therapeutic targets in psoriasis, with the inhibition of specific miRNAs presenting a promising approach for the development of novel treatment strategies (Nedoszytko et al., 2020). In conclusion, miRNAs make crucial contributions to the pathogenesis of psoriasis by regulating key processes, such as inflammation, cell proliferation, and cytokine responses. Understanding the alterations in miRNA expression levels that occur in response to different treatments can provide valuable insights into disease progression and therapeutic outcomes in psoriasis patients. Many miRNAs exhibit differential expression in response to psoriasis treatments, as shown in Table 1.

**TABLE 1:** Differential expression of miRNAs after psoriasis treatment.

miRNA	Tissue/cell	Treatment	Expression	Function	Reference
miR-206	Fibroblasts	Aminolevulinic acid PDT	Up	Promote apoptotic processes	Zhang et al. (2020)
miR-125	Fibroblasts	NB-UVB	Down	Inhibit axonogenesis	Nylander et al. (2011)
miR-21	Fibroblasts	NB-UVB	Down	Increase p63 expression, promoting apoptosis	Nylander et al. (2011)
miR-150	Keratinocytes	NB-UVB and methotrexate	Down	Increase HIF-1α and VEGFA expression	Li et al. (2017)
miR-203	Keratinocytes	NB-UVB and methotrexate	Up	Increase NR1H3 and PPADG expression	McKenna et al. (2010)
miR-149	Keratinocytes	NB-UVB and methotrexate	Up	Block the cross-talk between IFNG and TNFSF12	Srivastava et al. (2021)
miR-155	Keratinocytes	NB-UVB and methotrexate	Down	Decrease caspase-3 expression	Soonthornchai et al. (2021)
miR-4516	Lesional skin	PUVA therapy	Up	Decrease UBE2N expression, promoting apoptotic processes	Chowdhari and Saini (2014)
miR-29a	Lesional skin	Vitamin A and fractional laser	Down	Increase TGF-β, increasing the levels of collagen	Kocic et al. (2019)
miR-98	Lesional skin	Adalimumab	Up	Inhibit TNF-α	Raaby et al. (2015)
miR-214-3P	Lesional skin	Adalimumab	Up	Inhibit TNF-α	Raaby et al. (2015)
miR-125a-5p	Lesional skin	Adalimumab	Up	Inhibit TNF-α	Raaby et al. (2015)
let-7d-5p	Lesional skin	Adalimumab	Up	Inhibit TNF-α	Raaby et al. (2015)
miR-125a	Lesional skin	Adalimumab	Up	Inhibit TNF-α	Raaby et al. (2015)
miR-23b	Lesional skin	Adalimumab	Up	Inhibit TNF-α	Raaby et al. (2015)
miRNA-1290	Lesional skin	Adalimumab	Down	Inhibit TNF-α	Raaby et al. (2015)
miR-193	MSCs	Antagomirs and irradiation	Down	Decrease CDK2 & CCND1 expression	Wang et al. (2012)
miR-106b	Serum	Etanercept	Down	Inhibit TNF-α	Pivarcsi et al. (2013)
miR-26b	Serum	Etanercept	Down	Inhibit TNF-α	Pivarcsi et al. (2013)
miR-142-3p	Serum	Etanercept	Down	Inhibit TNF-α	Pivarcsi et al. (2013)
miR-223	Serum	Etanercept	Down	Inhibit TNF-α	Pivarcsi et al. (2013)
miR-126	Serum	Etanercept	Down	Inhibit TNF-α	Pivarcsi et al. (2013)
miR-128a	Serum	Methotrexate	Up	Immunosuppression	Jibing et al. (2024)
let-7d	Serum	Methotrexate	Up	Immunosuppression	Jibing et al. (2024)
miR-142-3p	Serum	Methotrexate	Up	Immunosuppression	Jibing et al. (2024)
miR-181a	Serum	Methotrexate	Up	Immunosuppression	Jibing et al. (2024)

MSCs, Mesenchymal stem cells; PDT, photodynamic therapy; NB-UVB, Narrowband ultraviolet B phototherapy; PUVA, Psoralen ultraviolet A photochemotherapy; HIF-1α, Hypoxia-inducible factor 1-alpha; VEGFA, vascular endothelial growth aactor a; NR1H3, Nuclear receptor subfamily 1 group H member 3; PPADG, Peroxisome proliferator-activated receptor gamma; IFNG, interferon gamma; TNFSF12, Tumor necrosis factor ligand superfamily member 12; UBE2N, ubiquitin-conjugating enzyme E2 N; TGF-β, Transforming growth factor beta; TNF-α, Tumour necrosis factor alpha; CDK2, cyclin-dependent kinase 2; CCND1, Cyclin D1.

### 5.3 Changes in exosomes after psoriasis treatment and its potential in therapy

Effective treatment in psoriasis patients induces significant modifications in extracellular vesicle composition and function. Therapeutic interventions of Methotrexate and NB-UVB consistently reduce pro-inflammatory exosomal miRNAs (miR-155, miR-31) while restoring protective miRNAs (miR-125b, miR-203), with miR-135b levels showing strong correlation to clinical improvement (Zhang et al., 2023). After systemic treatment with biologic agents such as anti-TNF-α or anti-IL-17/23 therapy, the concentration of exosomes in the serum and lesional tissues of patients significantly decreased compared to pretreatment levels, and this reduction was positively correlated with the levels of exosome-associated inflammatory factors (Paolino et al., 2022). Although studies on exosomal changes following psoriasis treatment remain limited, accumulating evidence has demonstrated significant differences in exosome profiles between psoriatic patients and healthy controls (Wang et al., 2018; Paolino et al., 2022; Zhang et al., 2022; Kim et al., 2023; Sun et al., 2023; Dehghani et al., 2024; Iuliano et al., 2024; Jibing et al., 2024; Yu et al., 2024). Given their unique structure and biological functions (Shi et al., 2021), exosomes, carrying disease-associated miRNAs like miR-21, miR-135b and proteins like S100A8/A9, IL-17 which hold promising potential as therapeutic targets or delivery vehicles for psoriasis (Guinea-Viniegra et al., 2014; Qiao et al., 2018; Chicharro et al., 2020). Emerging research suggests that engineered exosomes, either by modulating pathogenic miRNAs or loading anti-inflammatory cargo, could offer novel strategies for precision therapy. Further exploration of exosome-based interventions may bridge the gap in current treatment limitations, particularly for refractory cases.

Research has shown that exosomes derived from keratinocytes can activate neutrophils, leading to enhanced skin inflammation in psoriasis (Jiang et al., 2019; Zhang et al., 2021; Iuliano et al., 2024). Additionally, exosomes from psoriatic cells carry signaling molecules such as IL17, which can disrupt skin homeostasis and influence disease severity (Zhang and Wu, 2023). From a therapeutic perspective, exosomes from adipose-associated stem cells can alleviate inflammation induced by serum exosomes from psoriasis patients through the regulation of autophagy in keratinocytes (Kim et al., 2023). Furthermore, exosomes isolated from mesenchymal stem cells (MSCs) have been demonstrated to reduce psoriasis-like skin inflammation by modulating immune cells and keratinocytes (Zhang et al., 2022). Moreover, the use of exosomes for drug delivery in the context of psoriasis treatment has been investigated. For example, keratinocyte exosomes have been investigated for topical application of tofacitinib, a therapy for psoriasis, and have shown promising effects in both *in vitro* and *in vivo* models (Dehghani et al., 2024). Similarly, exosomal miR-4505 from vitamin D receptor-deficient keratinocytes promotes macrophage polarization towards the M1 phenotype, suggesting a role in modulating immune responses in psoriasis (Sun et al., 2023). Furthermore, topically applied MSC-derived exosomes have been shown to alleviate psoriasis-like inflammation, indicating their immunomodulatory properties (Zhang et al., 2021). In conclusion, exosomes play important roles in the pathogenesis and treatment of psoriasis, with implications for inflammation, immune modulation, and drug delivery. Understanding the mechanisms by which exosomes influence psoriasis will lead to novel therapeutic strategies for the management of this chronic skin condition.

### 5.4 Changes in the cytoskeleton and ECM in keratinocytes and fibroblasts after psoriasis treatment

In psoriasis, the interplay between keratinocytes and fibroblasts is important for disease pathogenesis and treatment response. Studies have highlighted specific differences in ceramide profiles between keratinocytes and fibroblasts in psoriasis patients, suggesting a role for lipid metabolism in the dermis and epidermis (Łuczaj et al., 2020). Birch bark triterpenes have been shown to induce shape changes via the actin cytoskeleton in both keratinocytes and fibroblasts, activating signaling pathway components such as Rho-GTPases and p38MAPK in keratinocytes (Wardecki et al., 2016). Ceramide has differential effects on cell growth and ECM reconstruction in keratinocytes and fibroblasts, impacting processes such as differentiation and ECM breakdown (Philips et al., 2009). Fibroblasts exhibit specific gene expression patterns in response to keratinocyte-released factors, influencing ECM synthesis and breakdown in the skin (Ghaffari et al., 2006; Clément et al., 2022). Changes in the organization of the actin cytoskeleton in epithelial cells and fibroblasts under restrictive conditions result in distinct behaviors, highlighting the importance of cytoskeletal dynamics in cell function (Jalal et al., 2019). Keloid fibroblasts are essential for ECM deposition and remodeling, providing mechanical stability and support for other cells during the wound healing process (Schneider and Wickström, 2015). Syndecan-1 and -4 expression alterations observed in psoriasis further emphasize the of cell‒cell communication between keratinocytes and fibroblasts in disease development (Peters et al., 2021). These findings collectively underscore the intricate interactions between keratinocytes and fibroblasts in psoriasis, which impact processes such as lipid metabolism, cytoskeletal dynamics, ECM remodeling, and cell signaling. Understanding the crosstalk between these cell types is essential for elucidating the pathogenesis of psoriasis and developing targeted therapeutic interventions.

## 6 Conclusion

The dysregulation of miRNAs and exosomes in psoriasis contributes to the disruption of cytoskeletal dynamics and ECM homeostasis, leading to the characteristic pathological features of the disease. Understanding the intricate interactions among miRNAs, exosomes, the cytoskeleton, and the ECM provides potential therapeutic targets for psoriasis intervention. Modulating miRNA expression or exosome release and cargo composition could restore cytoskeletal and ECM homeostasis, mitigating disease pathology. Furthermore, the development of targeted delivery systems for specific miRNAs or exosomes holds promise for localized and personalized treatments in the context of psoriasis management.

The intricate interplay among miRNAs, exosomes, cytoskeleton dynamics, and ECM remodelling in psoriasis highlights the essential roles of these components in disease pathogenesis. miRNAs and exosomes serve as critical mediators of cellular communication and regulatory networks, modulating key processes involved in psoriatic skin alterations. Further investigations into the functional roles, regulatory mechanisms, and therapeutic potential of miRNAs and exosomes in psoriasis will lead to a better understanding of the disease and the development of novel therapeutic strategies.

The use of miRNAs and exosomes as diagnostic biomarkers, prognostic indicators, and therapeutic targets has great potential for personalized medicine approaches for psoriasis. However, further research is necessary to validate the clinical utility of these factors, optimize delivery systems based on lipid nanoparticles, and evaluate their safety and efficacy in large-scale clinical trials.

Overall, it is important to elucidate the complex molecular mechanisms underlying cytoskeleton and ECM dysregulation in psoriasis and study the miRNAs, proteins, DNA, and other factors that are carried by exosomes and related to changes in the cytoskeleton and extracellular matrix in psoriasis. These approaches can assist in the analysis of the regulatory network of miRNAs, ceRNAs and proteins, thus providing insights into psoriasis, particularly when a biomarker candidate set has been identified in initial studies. Moreover, basic experimental research can provide bioinformatics data that approximate the *in vivo* situation. Thus, investigations of miRNAs, exosomes, the cytoskeleton and ECMs will reveal new opportunities for innovative therapeutic interventions and improved patient outcomes.

## Abbreviations

ECM, Extracellular matrix; ER, Endoplasmic reticulum; GO, Gene ontology; HLA-Cw6, Human leukocyte antigen-cw6; hTid-1S, Human homolog of the *drosophila* tumour suppressor l; LCs, Langerhans cells; lncRNA, Long non-coding RNA; miRNA, MicroRNA; MK2, Mitogen-activated protein kinase-activated protein kinase 2; MK5, Mitogen-activated protein kinase-activated protein kinase 5; MMPs, M10 matrix metalloproteinases; MSCs, Mesenchymal stem cells; NB-UVB, Narrow-band ultraviolet b phototherapy; ncRNA, Non-coding RNA; NF-κB1, Nuclear factor kappa b subunit 1; p38MAPK, p38 mitogen-activated protein kinases; PDT, Photodynamic therapy; PUVA, Psoralen ultraviolet a photochemotherapy; TGF-β, Transforming growth factor beta; TH17, T-helper Cell 17; TH22, T-helper Cell 22; TNF-α, Tumour necrosis factor alpha.
